# Localized tissue mineralization regulated by bone remodelling: A computational approach

**DOI:** 10.1371/journal.pone.0173228

**Published:** 2017-03-17

**Authors:** Marcelo Berli, Carlos Borau, Oscar Decco, George Adams, Richard B. Cook, José Manuel García Aznar, Peter Zioupos

**Affiliations:** 1 Facultad de Ingeniería, Universidad Nacional de Entre Ríos, Ruta 11, Oro Verde, Entre Ríos, República Argentina; 2 Departamento de Ingeniería Mecánica, Instituto de Investigación en Ingeniería de Aragón (I3A), Universidad de Zaragoza, Zaragoza, España; 3 Musculoskeletal & Medicolegal Research Group, Cranfield Forensic Institute, DA of the UK, Shrivenham, United Kingdom; 4 nCATS, University of Southampton, Highfield, Southampton, United Kingdom; University of Notre Dame, UNITED STATES

## Abstract

Bone is a living tissue whose main mechanical function is to provide stiffness, strength and protection to the body. Both stiffness and strength depend on the mineralization of the organic matrix, which is constantly being remodelled by the coordinated action of the bone multicellular units (BMUs). Due to the dynamics of both remodelling and mineralization, each sample of bone is composed of structural units (osteons in cortical and packets in cancellous bone) created at different times, therefore presenting different levels of mineral content. In this work, a computational model is used to understand the feedback between the remodelling and the mineralization processes under different load conditions and bone porosities. This model considers that osteoclasts primarily resorb those parts of bone closer to the surface, which are younger and less mineralized than older inner ones. Under equilibrium loads, results show that bone volumes with both the highest and the lowest levels of porosity (cancellous and cortical respectively) tend to develop higher levels of mineral content compared to volumes with intermediate porosity, thus presenting higher material densities. In good agreement with recent experimental measurements, a boomerang-like pattern emerges when plotting apparent density at the tissue level versus material density at the bone material level. Overload and disuse states are studied too, resulting in a translation of the apparent–material density curve. Numerical results are discussed pointing to potential clinical applications.

## Introduction

Bone provides support and protection to the body, stores minerals and also enables mobility. Moreover, it is able to adapt, in a remodelling process, to local mechanical demands by distributing the tissue mass in response to a daily stress field [1–6]. This results in an heterogeneously mineralized material [7–10] showing bone structural units of different mineral contents. The mass distribution in the bone can be traced by the apparent density (*ρ*_*app*_), which is the mineralized wet mass over the sample volume (*V*_*t*_) and by its material density (*ρ*_*mat*_) which is the mass over the volume occupied by the material itself (*V*_*b*_). Therefore, the difference between these two densities is a geometrical one and is due to the presence of pores. Bone, therefore, regulates both its apparent and material densities to different degrees and different effects, to answer the loading demands placed upon it. After skeletal maturity is reached these processes continue to allow for damage repair and this results in a highly heterogeneous bone matrix which has been shown independently to affect bone strength [11]. It is widely accepted that as much as 70–80% of the variation in bone strength and stiffness can be explained by apparent density alone [12], while the remaining 20–30% is a corollary of the finer modulation of bone material density itself.

The relationship between *ρ*_*app*_ and *ρ*_*mat*_, however, is elusive and not well-studied, but recently evidence has emerged that bone composition at tissue and material levels are linked as shown in the studies of Zioupos et al.[13]. These authors demonstrated a boomerang-like curvilinear pattern when plotting *ρ*_*app*_ vs *ρ*_*mat*_, showing an inflection point that suggests a natural separation in the behaviour of cortical and cancellous bone. The consequences of this are twofold: at a fundamental level the results alluded to a profoundly different mineralizing pattern in cortical and cancellous bone, and at a practical level the findings may have implications for the accuracy of CT (Computer Tomography) and QCT (Quantitative Computer Tomography) scans used for diagnoses, or for assigning bone properties in micro-Finite Element simulations [13–19].

Modelling the bone remodelling process by computational methods was widely performed by two main approaches: macroscopic and microscopic. The main difference between these approaches lies in the scale of modelling which in turn allows describing with more or less detail the inner architecture of the bone. Macroscopic models [6,20–23] were extensively used due to the reduced computational cost, and although requiring an extensive mathematical formulation, allowed simulation of the remodelling process of an entire bone. In these models, the volumes occupied by both pores and bone itself at every location of the tissue are described by means of continuum (average) variables computed into a representative volume (*V*_*t*_). Thus, the bone density at each *V*_*t*_ is actually the apparent density, which is the main variable that evolves in response to mechanical stimuli. In fact, the amount of tissue resorbed by osteoclasts and formed by osteoblasts are averaged quantities too, leading to an averaged bone volume, porosity and material density variations over time.

On the other hand, microscopic models [24–27] are able to describe in detail the internal architecture of the bone (microstructure), allowing thorough simulations of small bone samples but requiring higher computational costs. In these models, pores and bone tissue are treated as separate domains in the volume subject to simulation. Both osteoclasts and osteoblasts activities are explicitly modelled by predicting the effect of BMUs on bone surface over time, and the mineral evolution of every bone department can be traced not only in time but in space, so that the level of bone mineralization from the free surface to the core can be described.

**Fig 1:**
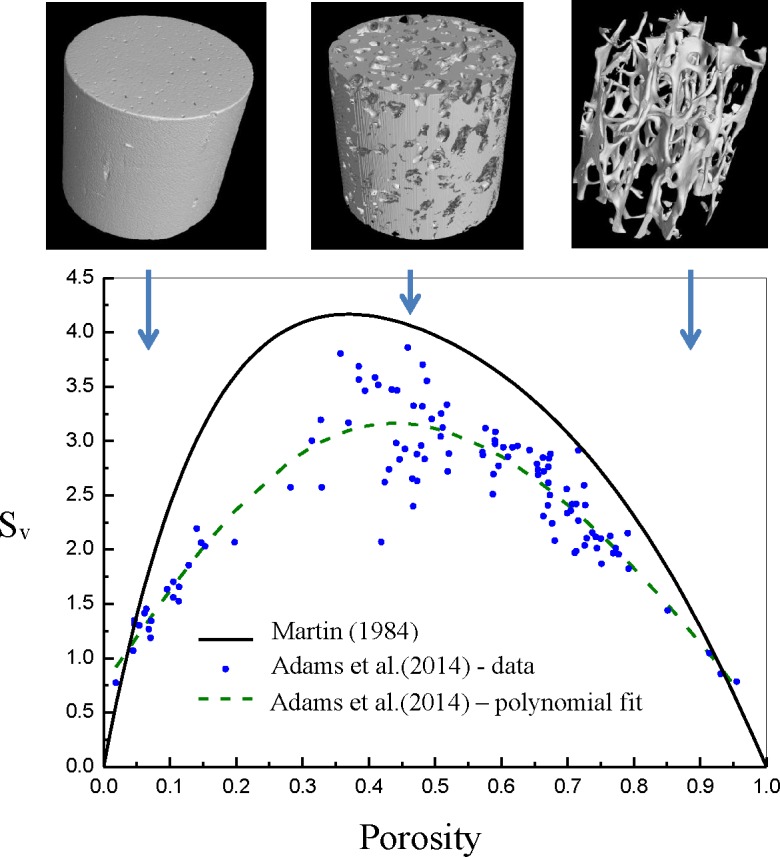
Specific Surface of bone as function of porosity (porosity = 1-BV/TV) from the analysis produced by Martin [30] (solid curve) and recent precise measurements by Adams et al.[28] (circles with a dashed line which is a fifth degree polynomial fit).

The amount of surface that is available depends of course, on the cellularity (level of porosity of this biological material. Martin (1984) has presented the parabolic curve of the specific surface of bone throughout the whole range from very compact (cortical) to least dense (cancellous) at the bottom end and showed that the maximum area available, through which remodelling acts, shows a maximum somewhere in between for porosity levels in the [0.3–0.6] range. Recent actual measurements of this phenomenon by members of this group showed that modern CT scanners can segment and produce these curves easily for a range of bones sample and from different species [28] (see Fig 1).

Several computational models dealing with the mineralization process of bone can be found in the literature, each one of them making different assumptions. For instance, Martínez-Reina et al.[21] developed a macroscopic model assuming that osteoclasts digest bone independently on their location by using averaged volumes. That is to say, the surface of bone (younger) was resorbed in the same manner as its core (older). This approach allowed tracking different bone variables in time (volumes, densities, mineralization) but was unable to consider geometrical effects. In this sense, Hartman et al.[27] implemented a microscopic stochastic model in which bone resorption was performed only on the bone surface but in a random manner not controlled by mechanical stimulus. Although they obtained realistic mineral density distributions, they concluded that some discrepancies of their results with respect to experiments may arise from the fact that osteoclasts resorb preferentially low mineralized young bone at the bone surface, something for which they did not account for in their model. Indeed, measurements on cancellous bone made by Lukas et al.[29] confirmed that bone gets more mineralized toward the core of the trabecula, suggesting that osteoclasts have an effect that depends on the depth from the surface, as if they have a tendency to resorb low mineralized bone at the trabeculae’s surface.

The present work is based on the fundamental approach that we established on macroscopic bone remodelling in the past [20,21], but now implementing a novel resorption strategy, which takes into account the observations that osteoclasts resorb preferably those parts of bone closer to the surface. This surface bone material is, in general, younger and less mineralized than the older inner one [29]. Further level of complexity is offered by exploring and considering the effects of different levels of initial bone densities, as well as external loads. The model is quantitatively compared with the trends observed in the relationship between *ρ*_*app*_ and *ρ*_*mat*_ under equilibrium state as published in Zioupos et al.[13,31]. Computational bone remodelling models are difficult to corroborate as they lack data for validation at the microstructural level. In this respect, the macroscopic experimental data of Zioupos et al.[13], provides a rare opportunity to verify how the mineral content is laid down and re-distributed within the bone and the effects it has on its local and global mineralization patterns. The aim of this paper is to show how the mineral distribution in bone, as evident in the recent work [13] is linked to this resorption strategy. This macroscopic approach constitutes then, a starting point to elucidate the biological mechanisms that could lead to the mineral distribution patterns observed in various other experimental observations.

## Bone-remodelling computational model

**Fig 2:**
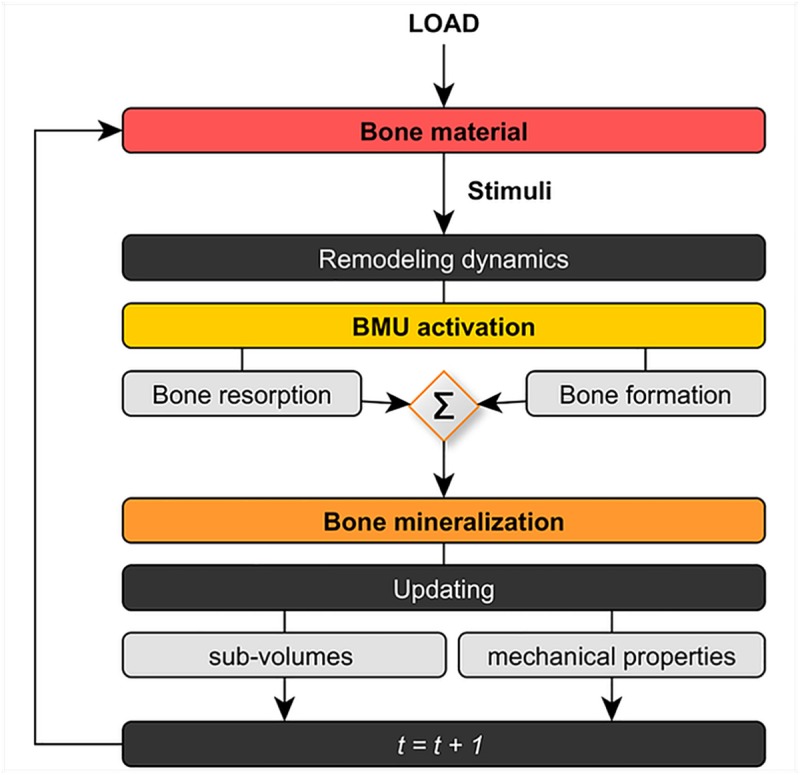
Simplified scheme of the bone-remodelling computational model [21]. Note that, for simplicity, some additional features like biological factors and damage dynamics are not shown in this flowchart to avoid confusion. Nonetheless, they are still present in the current approach.

The computational model proposed here is based on the same development steps introduced by García-Aznar et al.[20] and Martínez-Reina et al.[21]; these include a representative volume of bone, which is divided in different sub-volumes (solid matrix and pores) which evolve in time due to the action of the BMUs. BMUs constantly form and resorb bone activated by mechanical signals, which depend on external loads and material elasticity which in turn varies with bone mineralization (see Fig 2).

The present model incorporates a novel remodelling strategy based on the idea that osteoclasts tend to resorb newly formed bone closer to the surface, and not the highly mineralized one corresponding to the inner zones. Bone remodelling is a surface effect, very much a 2-D phenomenon which resembles diffusion in many of its details and its kinetics. This process and its implementation are detailed below; the main features that underpin the basic model are summarized in Appendix A.

### Bone composition

Bone tissue is composed by bone material and pores. The former is a composite of organic matrix (mainly collagen), water and mineral content, while the latter are filled with marrow, blood vessels and nerves. In this work, pores are regarded as void volumes, since the mechanical influence of the tissue inside is negligible with respect to the much stiffer matrix. Therefore, a reference bone tissue volume (*V*_*t*_) can be divided into the bone material volume (*V*_*b*_) and the volume of pores (*V*_*p*_). The bone material volume is in turn composed by sub-volumes of mineral phase (*V*_*m*_), organic phase (*V*_*o*_), and water (*V*_*w*_). Since bone is constantly being remodelled, every sub-volume evolves with time:

$$V_{t}=V_{b}\left(t\right)+V_{p}\left(t\right)=V_{m}\left(t\right)+V_{o}\left(t\right)+V_{w}\left(t\right)+V_{p}\left(t\right)$$ (1)

Note that *V*_*b*_ comprises volumes of portions of bone material formed at different times and therefore having a different mineralization level, as explained later.

To describe the temporal evolution of each sub-volume, the model defines the following ratios:

$$\begin{array}{cc} v_{b}(t)=\frac{V_{b}\left(t\right)}{V_{t}} & v_{m}(t)=\frac{V_{m}\left(t\right)}{V_{b}(t)}\\ v_{o}=\frac{V_{o}\left(t\right)}{V_{b}(t)} & v_{w}(t)=\frac{V_{w}\left(t\right)}{V_{b}(t)} \end{array}$$ (2)

We consider *v*_*o*_ to have a constant value of 3/7 [21], and water is assumed to be replaced by mineral during the mineralization process. Hence, the next sum holds:

$$v_{m}\left(t\right)+v_{o}+v_{w}\left(t\right)=1$$ (3)

Having the evolution of every component of bone material volume fraction, we can compute the material density *ρ*_*mat*_, which is actually the density of the solid bone matrix:

$$\rho_{mat}\left(t\right)=\rho_{m}\;v_{m}\left(t\right)+\rho_{o}\;v_{o}+\rho_{w}\;v_{w}\left(t\right)$$ (4)

Where densities of mineral, organic components and water are *ρ*_*m*_ = 3.2 g / cm^3^, *ρ*_*o*_ = 1.1 g / cm^3^ and *ρ*_*w*_ = 1.0 g / cm^3^ respectively [21]. This definition leads to a linear relationship between material density and mineral volume fraction. On the other hand, the apparent density *ρ*_*app*_, the basic variable in most bone remodelling models [2,3,5,32,33], can be obtained through the bone material volume fraction and the material density:

$$\rho_{app}\left(t\right)=\rho_{mat}\left(t\right)v_{b}\left(t\right)$$ (5)

The ash fraction, which is defined as the ratio between the mineral mass and the dry mass (the sum of mineral and organic mass) can be expressed as follows:

$$\alpha =\frac{\rho_{m}\;v_{m}}{\rho_{m}\;v_{m}+\rho_{o}\;v_{o}}$$ (6)

In this way, the temporal evolution of *v*_*m*_(*t*) is linked to changes of *α* and vice versa.

### Remodelling dynamics

**Fig 3:**
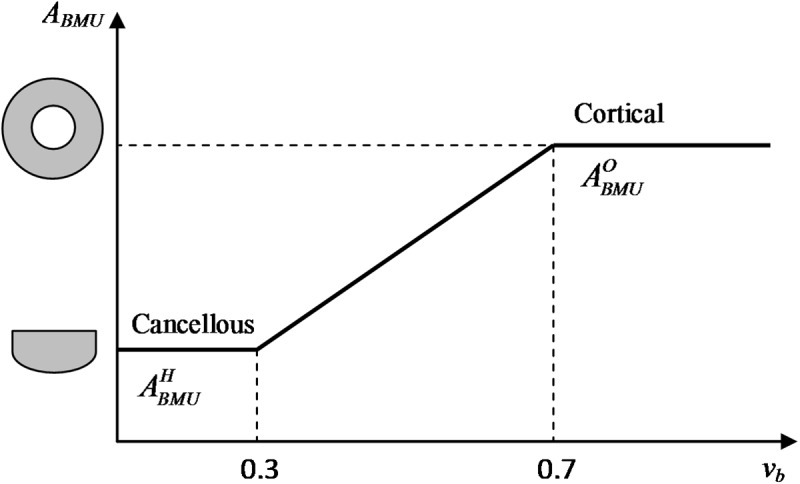
Cross section area (*A*_*BMU*_) of the tissue volume unit that a single BMU remodels in cortical and cancellous bone as a function of bone material volume. For *v*_*b*_ > 0.7, osteonal cross section is considered while for *v*_*b*_ < 0.3, hemi-osteonal cross section is used instead. For bone material volume values between 0.3 and 0.7, a linear transition of cross section area is assumed.

According to our previous model [21], as time advances, certain parts of bone material are formed (*v*_*f*_) while others are resorbed (*v*_*r*_) therefore updating the bone material volume at each time step (*v*_*b*_). Hence, the rate of change of the bone material volume fraction (${\dot{v}}_{b}$), depends on the volume of bone material removed (${\dot{v}}_{r}$) and formed (${\dot{v}}_{f}$) per unit time, by all BMUs active at time *t*. Therefore ${\dot{v}}_{b}\left(t\right)={\dot{v}}_{f}\left(t\right)-{\dot{v}}_{r}\left(t\right)$, with:

$${\dot{v}}_{r}\left(t\right)=\overset{t}{\underset{t-T_{R}}{\int }}\left(\overset{t´}{\underset{t´-\sigma_{L}}{\int }}{\dot{N}}_{BMU}\left(t´´\right)dt´´\right)\frac{A_{BMU}}{T_{R}}f_{c}\left(t^{\prime }\right)v_{BMU}dt´´$$ (7)

$${\dot{v}}_{f}\left(t\right)=\overset{t-T_{R}-T_{I}}{\underset{t-T_{R}-T_{I}-T_{F}}{\int }}\left(\overset{t´}{\underset{t´-\sigma_{L}}{\int }}{\dot{N}}_{BMU}\left(t´´\right)dt´´\right)\frac{A_{BMU}}{T_{R}}f_{b}\left(t^{\prime }\right)v_{BMU}dt´´$$ (8)

where ${\dot{N}}_{BMU}$ stands for the number of BMUs activated per unit time and unit volume (whose calculation can be consulted in appendix A), *v*_*BMU*_ is the speed of BMU progress, *T*_*R*_ is resorption period (first period of BMU action due to osteoclasts activity) followed by a reversal time (*T*_*I*_) after which deposition of bone is performed by osteoblasts during the formation period (*T*_*f*_), and *σ*_*L*_ is the lifespan of BMU. On the other hand, *f*_*c*_ and *f*_*b*_ measure the osteoclast/osteoblast activities respectively. It is important to note that the ratio *f*_*b*_/*f*_*c*_ follows a piece-wise linear model as a function of the unbalanced stimulus *ξ*−ξ*, being ξ* the reference stimulus (see [20]). If *ξ* = ξ*, *f*_*b*_ = *f*_*c*_, ${\dot{v}}_{r}\left(t\right)={\dot{v}}_{f}\left(t\right)$ and ${\dot{v}}_{b}\left(t\right)=0$ (equilibrium). For higher stimulus, *ξ* > ξ*, *f*_*b*_ >*f*_*c*_ and bone formation dominates (porosity decreases), while for disuse, *ξ* < ξ*, *f*_*b*_<*f*_*c*_ and resorption dominates (porosity increases). Finally, *A*_*BMU*_ is the section of the material volume unit resorbed by osteoclasts and then filled by osteoblasts. Osteon cross section is considered for the assumed range of cortical bone (*v*_*b*_ > 0.7), while hemiosteon is considered for cancellous bone (*v*_*b*_ < 0.3) [20]. In the transition zone, where 0.3 < *v*_*b*_ < 0.7, a linear approach of the BMU section *A*_*BMU*_ is assumed (Fig 3).

### Mineralization process

During the mineralization process, water is replaced by mineral and thus, the mineral content of every part of bone material increases with time [34]. As a result, the mineral volume of each part ($v_{m}^{*}$) evolves with time following the next assumed law (Eq 9), which distinguishes the three phases of mineralization: lag time where no deposition of mineral occurs, primary phase with a linear increase of mineral content, and the secondary phase with an exponentially decreasing rate [21]:

$$v_{m}^{*}(t)=\{\begin{array}{ll} 0 & if\;t\leq T_{nm}\\ v_{{}_{m}}^{prim}\frac{t-T_{nm}}{T_{prim}} & if\;T_{nm}<t\leq \left(T_{nm}+T_{prim}\right)\\ v_{{}_{m}}^{\max}-(v_{{}_{m}}^{\max}-v_{{}_{m}}^{prim})e^{-\kappa_{m}(t-T_{prim}-T_{nm})} & if\;\left(t_{nm}+T_{prim}\right)>t \end{array}$$ (9)

where *T*_*nm*_ and *T*_*prim*_ are the length of the mineralization lag time and the primary phase respectively and *κ*_*m*_ is a parameter which measures the rate of mineral deposition in the secondary phase. $v_{{}_{m}}^{prim}$ is a constant representing the mineral volume ratio at the end of the primary phase($v_{{}_{m}}^{prim}=0.121$) and $v_{{}_{m}}^{\max}$ corresponds to a volume ratio with the maximum calcium content ($v_{{}_{m}}^{\max}=0.442$, 300 mg/g) [35].

To compute the mineral volume fraction of the whole bone (*v*_*m*_), the mineralization law must be first established. The bone material volume fraction is composed by differential of bone created at day *τ* and still present at time *t*, this is:

$$v_{b}(t)=\int_{t-T_{m}^{Max}}^{t}\frac{dv_{b}(\tau )}{d\tau }d\tau$$ (10)

where $T_{m}^{Max}$ is the time required for a part of bone material to reach the maximum mineralization level. With every time increment, ${\dot{v}}_{b}(t)$ must be updated considering the amount of bone material formed ($dv_{f}(t)={\dot{v}}_{f}(t)dt$) and resorbed ($dv_{r}(t)={\dot{v}}_{r}(t)dt$). Normally, macroscopic bone remodelling laws do not take into account how and where this resorption occurs, but this is actually the cornerstone of the current approach. Our model assumes that osteoclasts tend to remove bone primarily from young surface areas, but only those presenting a minimum amount of mineral content, which excludes just newly created bone parts. Thus, we define a temporal window (Δ*τ* = *T*_*sr*_) that includes those parts of bone candidates to be resorbed. If we call the lower bound of this window the non-mineralization time (*T*_*nm*_), then the parts of bone material subject to be digested are those created in the time interval determined between *T*_*nm*_ and *T*_*nm*_ + *T*_*sr*_ days ago. Following this, *v*_*m*_(*t*) can be computed as:

$$\begin{array}{l} v_{m}(t)=\frac{1}{v_{b}(t)}\overset{t}{\underset{t-T_{m}^{Max}}{\int }}v_{m}^{*}\left(t-\tau \right)\frac{dv_{b}(\tau )}{d\tau }d\tau =\\ \;=\frac{1}{v_{b}(t)}\overset{t}{\underset{t-T_{m}^{Max}}{\int }}v_{m}^{*}\left(t-\tau \right)\frac{dv_{f}(\tau )}{d\tau }d\tau -\frac{1}{v_{b}(t)}\overset{t-T_{nm}}{\underset{t-T_{nm}-T_{sr}}{\int }}v_{m}^{*}\left(t-\tau \right)\frac{dv_{r}(\tau )}{d\tau }d\tau \end{array}$$ (11)

where $v_{m}^{*}(t-\tau )$ is the mineral volume fraction of each bone material part, calculated according to Eq 9. The computational implementation the mineralization process is discussed in the following section.

### Numerical implementation

**Fig 4:**
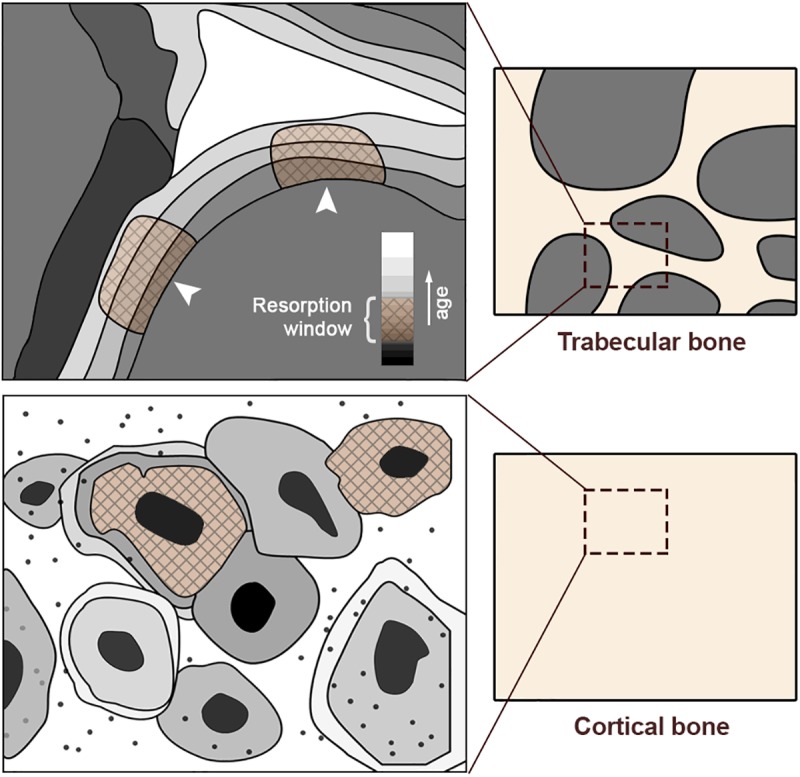
Plot representing a reference bone volume (right) formed by solid matrix and pores. A magnifying box honing in on specific trabeculae (left) shows a mosaic of tissue structural units presenting a distribution of mineral content depending on bone age (grayscale, darker means younger and less mineralized). Since resorption is assumed to be performed preferably at the bone surface, a resorption temporal window is defined (shaded areas with stripes). The organization of the trabeculae in layers with growing mineral content is based on results by Lukas et al.[29]. Also, there is experimental evidence of the layer-like organization in compact bone as can be seen in the work by Boivin et al.[36]

The algorithm for bone remodelling was implemented in a user material subroutine (UMAT) linked to Abaqus. The main purpose of the model is to track the evolution of the different sub-volumes comprising a representative bone material volume fraction to study the mass distribution (densities) once the system reaches equilibrium. In order to take into account that the mineral content of every part of bone material depends on modelling history, the time at which such parts were formed is also stored and used in the equations. Fig 4 shows a representation of the bone as a mosaic of bone material parts with different mineral contents. It must be noted that the representation of the bone material by layers of increasing mineral content is based on the results of Lukas et al [29].

Since the mineralization could, theoretically, evolve indefinitely (see Eq 9), we define a maximum mineralization time ($T_{m}^{Max}$) at which a certain volume is considered to reach $v_{{}_{m}}^{\max}$. In this way, we set a sufficiently large window data while keeping down the computational costs (array lengths) (see Table 1).

**Table 1:** Parameters used in the simulations, extracted from [21].

Parameter		Reference	Range
*General parameters*			
$A_{BMU}^{O}$	Cortical *A*_*BMU*_	4.3x10^-3^ mm^2^	
$A_{BMU}^{H}$	Cancellous *A*_*BMU*_	1.9x10^-3^ mm^2^	
*f*_*c*_	Measurement of osteoclast activity	1.0	
*f*_*b/*_*f*_*c*_	Balance between osteoblasts and osteoblasts activity.	1.0	0.95–1.05
ξ*	Reference stimulus	0.00025	0.0–0.0005
*ν*	Poisson coefficient	0.3	
*v*_*o*_	Specific volume of organic phase	3/7	
*ρ*_*m*_	Density of mineral phase	3.2 g/cm^3^	
*ρ*_*o*_	Density of organic phase	1.1 g/cm^3^	
*ρ*_*w*_	Density of water	1.0 g/cm^3^	
*Time spams in BMU activity*			
*T*_*r*_	Resorption period	24 days	
*T*_*I*_	Reversal period	8 days	
*T*_*f*_	Formation period	64 days	
*v*_*BMU*_	Speed of BMU progress	0.04 mm/day	
*σ*_*L*_	Lifespan of BMU	100 days	
*Mineralization*			
*T*_*nm*_	Mineralization lag time	12 days	
*T*_*prim*_	Length of primary phase	10 days	
*$T_{m}^{Max}$*	Time to reach the maximum mineral level	4000 days	
$v_{{}_{m}}^{prim}$	*v*_*m*_ at the end of primary phase	0.121	
$v_{{}_{m}}^{\max}$	Maximal mineral specific volume	0.442	
*κ*_*m*_	Rate of secondary phase	0.0005	
*κ*_*sr*_	Scaling parameter controlling the size of the resorption window	200 days	150–250

To account for the evolution of every part of bone material, the bone material volume fraction (*v*_*b*_) is computationally implemented in an array containing the discrete portions of bone material volume fractions Δ*v*_*b*_(*i*), where every component of this array is the part of bone material formed *i* days ago and still present at time *t*. This array is homogeneously initialized in each simulation as:

$$\Delta v_{b}\left(i\right)=v_{b}^{0}/T_{m}^{Max}\;\text{for}\;0<i\leq T_{m}^{Max}$$ (12)

where $v_{b}^{0}$ is the initial bone material volume fraction considered in each computation, ranging from 0.2 (cancellous bone) to 0.94 (cortical). How each component of Δ*v*_*b*_(*i*) is calculated and updated is explained below in this section and is the essential foundation of our model.

Firstly, from Eq 11 we distinguish four temporal windows: 1) the old mineralized tissue (formed between *T*_*sr*_ + *T*_*nm*_ and $T_{m}^{Max}$ days ago), far from the pore surface, 2) the younger bone material that is more likely to be resorbed (formed between *T*_*nm*_ and *T*_*sr*_ + *T*_*nm*_ days ago), 3) the recently created bone material that lacks mineral content and is assumed to be ignored by the osteoclasts (formed from the current day to *T*_*nm*_ days ago) and 4) the recent bone material added at present time *t* due to osteoblasts activity. Hence, considering a computational time step of 1 day (i.e. Δ*t* = 1 day), the bone volume fraction at each step is computed following Eq 13:

$$v_{b}(t)=\overset{1)}{}\sum_{i=T_{nm}+T_{sr}}^{T_{m}^{Max}}\Delta v_{b}(i)+\overset{2)}{}\sum_{i=T_{nm}}^{T_{nm}+T_{sr}-1}\left[\Delta v_{b}(i)-\Delta v_{r}(i)\right]+\overset{3)}{}\sum_{i=1}^{T_{nm}-1}\Delta v_{b}(i)+\overset{4)}{}\Delta v_{f}(t)$$ (13)

where Δ*v*_*r*_(*i*) is the array containing the portions to be removed from the parts of bone material formed *i* days ago, whose summation must fulfil that:

$$\sum_{i=T_{nm}}^{T_{nm}+T_{sr}-1}\Delta v_{r}(i)={\dot{v}}_{r}(t)\Delta t$$ (14)

**Fig 5:**
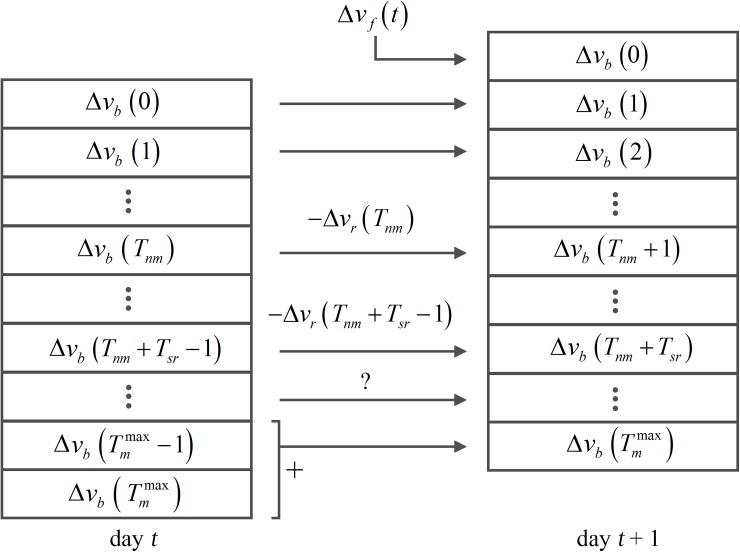
Updating scheme for the bone volume fraction array (Δ*v*_*b*_) of length ($T_{m}^{Max}+1$). The resorption starts on those components between *T*_*nm*_ and *T*_*nm*_ + *T*_*sr*_ − 1 positions. Note that if the values stored in such positions are not enough to fulfil the resorption rate at a specific time, further components are removed until the corresponding amount of bone is resorbed (question mark). Bone material surpassing the maximum mineralization time ($T_{m}^{max}$) is stored at the last position of the array to minimize computational costs.

It is worth noting that we assume that the volume to be resorbed is uniformly distributed along its *i* components, which means that the amount of bone material resorbed at time *t* takes the same number of parts from different ages along the resorption window ($\Delta v_{r}(i)={\dot{v}}_{r}(t)\Delta t/T_{sr}$). It is also important the fact that in case that there is not enough bone material of a certain age to be removed, the resorption spreads to older parts of bone material, which is especially relevant in cases of disuse where ${\dot{v}}_{r}>{\dot{v}}_{f}$. This process is schematized in Fig 5.

Importantly, to account for the osteoclast population (and hence BMU activity) depending on the type of bone, we assume that the resorption window size varies proportionally to the available bone material specific surface (*S*_*v*_), therefore depending on porosity (*p* = 1−*v*_*b*_) in a curvilinear parabolic fashion [28, 30]:

$$T_{sr}=\kappa_{sr}S_{v}=\kappa_{sr}\left(28.76p^{5}-101.4p^{4}+133.96p^{3}-93.94p^{2}+32.26p\right)$$ (15)

where *κ*_*sr*_ is a scaling parameter to control the window resorption size. In this way, bone with very high or very low porosity (less surface area) is assigned smaller resorption windows compared to bone of intermediate porosity. Consequently, this fact alters the outcome of the mineralization process, because, and over a period of time, inner unresorbed parts of bone material with very low/high porosity keep on increasing their mineral content compared to bone in the transition from cancellous to cortical bone.

Once *v*_*b*_ is updated following the scheme shown in Fig 5, the new fraction of mineralized bone can be computed as follows:

$$v_{m}\left(t\right)=\frac{1}{v_{b}(t)}\overset{T_{m}^{max}}{\underset{i=0}{\sum }}\Delta v_{b}\left(i\right)v_{m}^{*}\left(i\right)$$ (16)

Consequently, *v*_*w*_ can be isolated and therefore *ρ*_*mat*_ and *ρ*_*app*_ can be obtained from Eqs 3 to 5. Further details about the mechanical problem and the computation of the stimulus driving the bone formation/resorption can be found in S1 Text.

## Results and discussion

In order to test the impact of this bone’s resorption hypothesis we examine the values that it gives us for material and apparent densities (*ρ*_*mat*_, *ρ*_*app*_) after a period of more than 20 years, having several initial bone material volume ratios, and different load cases. In particular, we explore the evolution of bone material with $v_{b}^{0}$ ranging from 0.2 to 0.94 and three levels of external load, namely: *ξ* = ξ* (equilibrium), *ξ* = 0 (disuse) and *ξ* = 2ξ*(overload). To facilitate the discussion, we will differentiate between three zones depending on the initial bone volume ($v_{b}^{0}$): (i) cancellous ($v_{b}^{0}<0.3$); (ii) transition ($0.3<v_{b}^{0}<0.7$) and (iii) cortical ($v_{b}^{0}>0.7$).

### Resorption dynamics regulates bone mineral distribution

Although it is well known that osteoclasts remove bone starting from the surface, how this phenomenon regulates bone mineralization is not completely understood. In this section, three different ways of resorption are tested, and the resulting material and apparent densities (*ρ*_*mat*_, *ρ*_*app*_) are compared with experimental measurements. In case-1, we use the assumptions from the previous model [21], in which osteoclast resorption is homogeneously distributed and independent of the bone level of mineralization (or equivalently, age). In case-2, osteoclasts are allowed to remove only the newly formed and poorly mineralized -bone material. In case-3, resorption is performed in the way proposed in this work, that is, being limited to a temporal window covering from recently, but not just newly created tissue, to those parts of bone material with an intermediate level of mineralization (formed between *T*_*nm*_ and *T*_*sr*_ + *T*_*nm*_ days ago). To analyse the effect of the resorption window size (*T*_*sr*_), different values of *κ*_*sr*_ are explored (see Eq 15). All simulations in this section are performed under an equilibrium load *ξ* = ξ*.

**Fig 6:**
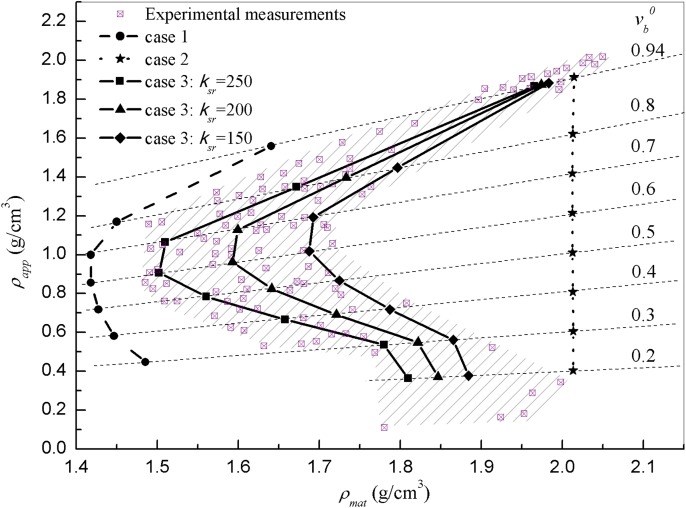
Tissue density (*ρ*_*mat*_) vs apparent density (*ρ*_*app*_) of bone for different temporal resorption conditions (initial bone volumes $v_{b}^{0}$ ranging from 0.2 to 0.94) superimposed with experimental measurements [13]. The plot presents the values at equilibrium state (*ξ* = ξ*, *t* = 8000 days). Case-1 (dashed line with circles): resorption of bone is independent on temporal history and it is averaged through all the volume, including the most mineralized sub-volumes. This leads overall to the lowest material tissue densities for all the starting conditions we examine. Case-2 (dotted line with stars): resorption is only allowed to happen at the newly created bone, collapsing the curve to a vertical line where all bone volume is fully mineralized regardless $v_{b}^{0}$. Case-3: resorption is limited to temporal windows (bone parts created between *T*_*nm*_ and *T*_*sr*_ + *T*_*nm*_ days ago), with three values of *κ*_*sr*_: 150 (solid line with diamonds), 200 (solid line with triangles) and 250 (solid dashed line with squares). The introduction of the hypothesis that resorption applies not just on newly created bone compartments but to recently formed bone up to 250 days, shifts the curves producing an envelope which practically covers the complete experimentally observed data set of points (cross-square dots).

Fig 6 shows the simulated results superimposed to experimental measurements from [13]. In case-1 (dashed line with circles) all points fall at the left and outside of the measurements area, that is, the bone material presents lower *ρ*_*mat*_ than the experiments for all conditions (regardless of the initial $v_{b}^{0}$). This is due to the process being averaged over the complete bone material volume. Since the osteoclasts are able to reach the core of the bone material volume where those bone structural unit with the highest mineral content reside, the model predicts a lower mineralized tissue compared to the experimental data. Case-2 (dotted line with stars) represents the completely opposite idea: only those surface layers recently formed can be removed, so that the inner bone material remains unaltered and therefore has more time to reach higher mineralization levels. This hypothesis is too drastic and after a certain time, all the material is fully mineralized. This assumption yields a constant *ρ*_*mat*_ value regardless of the value of *ρ*_*app*_ (resulting in a vertical line) and predicts higher material densities for all the studied cases (right side of measurements data). These results, together with those from case-1, suggest that some hypothesis in between, like that proposed in case-3, might be adequate to match the experiments. In fact, results confirm an excellent agreement between the simulations from case-3 (solid lines with different markers) and the experimental measurements. Lower values of *κ*_*sr*_ lead to higher mineralization levels (higher *ρ*_*mat*_) while higher values of *κ*_*sr*_ produce low mineralized bones. Thus, varying *κ*_*sr*_ allows a gradual shifting of the curves to the desired levels of densities. Note that if *κ*_*sr*_ was big enough to make *T*_*sr*_ as large as $T_{m}^{Max}$ (the length of the array storing the history of bone material volumes), case-3 would be equivalent to case-1, since all the bone material would be subject to resorption regardless of its age and mineralization level.

In all cases, for all the values of *κ*_*sr*_ studied, the *ρ*_*mat*_−*ρ*_*app*_ curves show a boomerang-like pattern as that shown by Zioupos et al.[13]. This shape can be explained by the model assumptions themselves. Based on the model predictions, the adaptation of osteoclasts activity seems to depend on the available surface and therefore on porosity (or equivalently on *v*_*b*_). Both cortical and cancellous bones (presenting lowest and highest porosities respectively), have small available surfaces so that the resorption concentrates on few portions of bone, which are constantly being renewed, while the inner parts keep mineralizing. This results in bones with very high *ρ*_*mat*_ and very high/low *ρ*_*app*_ (cortical/cancellous) which form the two tale ends of the boomerang pattern. On the other hand, bones with porosities in the transition zone ($0.3<v_{b}^{0}<0.7$) have higher available surfaces so that the resorption reaches portions of bone with several levels of mineralization. This decreases the overall mineral content and thus forms the “nose” of the boomerang.

It is worth making here a comment on the absolute values of material density (*ρ*_*mat*_). These at the peak of the curve at their lowest show values in the range of [1.5–1.7] g/cm^3^, which perhaps appear too low when according to Martin [30] osteoid has a density of 1.41 g/cm^3^ and fully mineralized bone has a density of 2.31 g/cm^3^. However, these (*ρ*_*mat*_) were produced by the Archimedes suspension technique for measuring material density and this method thresholds everything that has a density higher than the suspending medium (1 g/cm^3^ for water). For Archimedes ‘osteoid’ starts at the collagen level (density of about 1.06 g/cm^3^ or thereabouts) and everything above that is ‘bone’. Radiographers and all other researchers define bone where mineral deposition has started and has actually taken place in a noticeable way. This indeed is for densities of 1.41 g/cm^3^ and above, with some micro-CT scanners thresholding bone by default at 1.3 g/cm^3^. Hence in reality Archimedes encompasses a broader range of ‘bone’ than any radiographic perception of this tissue.

It is worth mentioning that some curve shifting could also be achieved by adding the impact of the residual fat that could affect experimental measurements of densities as was pointed out by Schileo et al.[37]. However, the boomerang-like pattern will still appear since it depends on the resorption kinetics and not on the actual density values, as was predicted by Zioupos et al.[31]. Therefore, although the precise location of the boomerang curve may be disputed, its very shape is undeniable. On the other hand, care must also be taken in the definition of densities when analysing the results. For instance, if the material density is replaced by the ash density (*ρ*_*ash*_) defined as the density of the mineral mass (*ρ*_*m*_*V*_*m*_) divided by the total bone tissue volume (*V*_*t*_), a near monotonic trend of the *ρ*_*mat*_ vs *ρ*_*ash*_ relationship is obtained, which is in accordance with the experimental measurements by Schileo et al.et al.[38] (S1 Fig).

### External loads determine bone formation and mineralization

The very essence of mechanobiology is the response of tissues to external mechanical stimuli. So far, the effects of resorption dynamics have been explained under a specific load condition of *ξ* = ξ* (equilibrium). In this section, we compare such effects with those produced by two diametrically opposed cases: overload (*ξ* = 2ξ*) and disuse (*ξ* = 0).Overload is related to bone material changes during intense activity, whereas disuse is associated to prostration, microgravity and sedentary lifestyle, among others.

**Fig 7:**
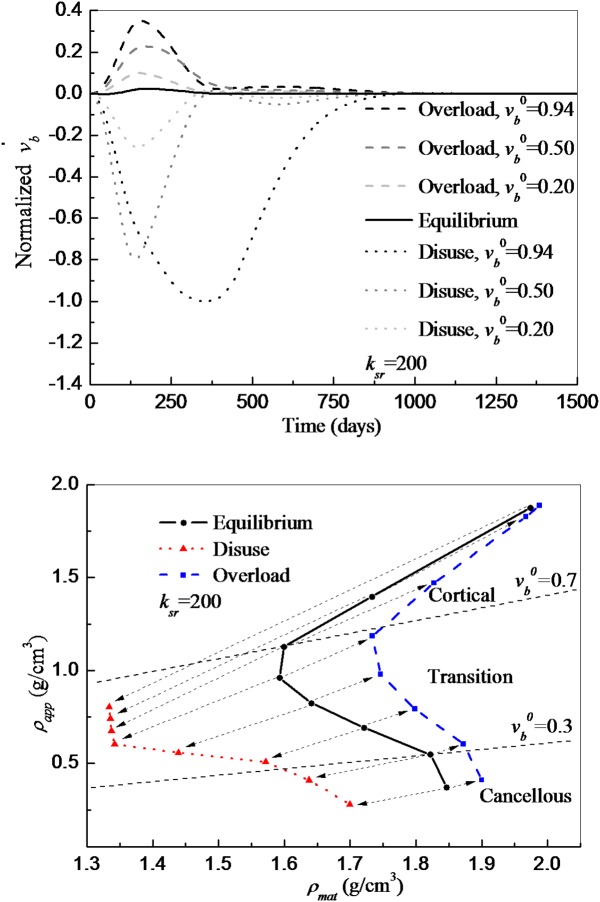
(A) Normalized bone volume formation rate for different load conditions (*ξ* applied uniaxial) and initial bone volumes ($v_{b}^{0}$). A parameter of resorption value of *κ*_*sr*_ = 200 was used in all the cases. Equilibrium (*ξ* = ξ*, solid line): the amount of bone formed is the same as that of resorbed, leading to ${\dot{v}}_{b}=0$ with only small oscillations during the first 400 days. Disuse(*ξ* = 0, dotted lines): the amount of bone resorbed is much higher than bone being formed, leading to negative ${\dot{v}}_{b}$ until reaching equilibrium(${\dot{v}}_{b}=0$) after about 1000 days. Overload (*ξ* = 2ξ*, dashed lines): the amount of bone formed is higher than bone being resorbed, leading to positive ${\dot{v}}_{b}$ until reaching equilibrium (${\dot{v}}_{b}=0$) after about 1000 days. In both cases, the impact is especially relevant during the first 200 days in which the bone composition exhibits strong variations. After reaching a minimum/maximum, the effect gradually disappears, with small oscillations until equilibrium. (B) Tissue density (*ρ*_*mat*_) vs apparent density (*ρ*_*app*_) of bone for different load conditions (*ξ* Pa applied uniaxial). Arrows show translation of points with the same initial bone volume(*v*_*b*_) from the equilibrium case (solid line with circles) to disuse or overload. In disuse (dotted line with triangles), both *ρ*_*mat*_ and *ρ*_*app*_ are greatly reduced compared to the equilibrium case. In the case of overload (dashed line with squares), *ρ*_*app*_ is mainly conserved, whereas *ρ*_*mat*_ is increased especially in the transition zone (0.3 < *v*_*b*_ < 0.7).

As shown in Fig 7A, overload leads to an enhanced bone material formation, especially relevant during the first 200 days, until the steady state (equilibrium) is reached after 1000 days. This net formation has a direct effect on mineralization since the inner parts are less likely to be resorbed and therefore their mineral content rises in time. This results in a lateral shift of the *ρ*_*mat*_−*ρ*_*app*_ curve towards overall higher material densities (right). This shift is greater at the transition zone where the bone kinetics is more intense (greater specific surface) and rapidly reduces in the cortical one. The observed shift has two corollaries: bone would appear to become more uniform in material density values and would also be perceived to be reinforced to support higher loads as has been demonstrated in various experiments [39,40].

By contrast, disuse cancels the inhibitory signal leading to an enhanced BMU activity which in turn creates an imbalance that greatly favours osteoclasts. The higher resorption activity leads to a dramatic reduction of bone volume (mainly in cortical bones) allowing osteoclasts to resorb greater amounts of high mineralized bone material and therefore substantially decreasing *ρ*_*mat*_, which is in agreement with the experimental observations from [41]. Hence, all the points on the densities curve trajectory move to the left of the equilibrium case towards zones of less dense tissue (Fig 7B).

It must be noted that, under the almost fictitious extreme condition of complete disuse, “non-needed” bone is totally resorbed so that the cortical zone disappears. In this situation, the most mineralized bone material would be located in the trabecular zone. In turn, this means that physiologically, bone subjected to a long period of disuse would become so fragile that return to normal activities should be accomplished gradually and at pace dictated by the strength of the material. Although in reality this is not expected to happen throughout the whole bone, this phenomenon has been observed around implants supporting most of the load [42].

## Conclusions

Understanding the full gamut of effects that govern bone formation and resorption, and being able firstly to handle the various factors and secondly to predict likely outcomes via a comprehensive model is a long term goal for bone mechanobiology [43–46]. The present work provides a novel paradigm in this quest by formulating our basic understanding of the material on some new basic assumptions on the spatial and temporal process of bone mineralisation in-situ and then validating it against some very recent extraordinary observations on the way bone densities behave locally and globally across the full range of bone volumes from cortical to cancellous [13,31]. The model was based on the earlier work by [20,21] and introduces a novel mathematical scheme implementing the bone formation/resorption cycle. Numerical predictions of this computational approach for the remodelling process are notoriously difficult to validate because the observations on determination of the spatial distribution of mineral content in the bone are, by and large, qualitative in their nature deriving from microscopy studies. However, the data presented by Zioupos et al.[13,31], on the macroscopic relationship between apparent density *ρ*_*app*_ (at tissue level) and material density *ρ*_*mat*_ (at material level) is truly quantitative and allows for this, so elusive, validation of the model.

Model predictions suggest a strong relationship between remodelling dynamics and the boomerang-like pattern of the *ρ*_*app*_−*ρ*_*mat*_ curve, with osteoclasts playing a key role in determining the mineral distribution in bone. Their activity has been linked to the available surface (remodelling is a 2-D effect driven and mediated through an active surface area) and therefore to porosity because the surface density of a cellular solid goes up and down with the overall porosity. In this sense, the model further stipulates that bone resorption happens preferentially on the younger low mineralized tissue, but after a minimal amount of mineral is reached. A geometric effect in the sense of depth from the free surface is also implicitly part of the formulation because newly formed less mineralised bone is more towards the surface with more mature and mineralised bone buried deep in the inner cores of the trabeculae. A suitable choice of the resorption window allows achieving accurate computational predictions and permits the adaptation of the model to account for the variability of the experimental data.

Mechanical loading also plays its part in determining the mineral distribution through the bone structure. Overload enhances bone formation, reinforcing the bone to support higher loads. Bone volume is increased at the surface so that the inner tissue increases even more its mineral levels, leading to higher *ρ*_*mat*_ compared to the equilibrium. It must be clarified that the selected load for this case(*ξ* = 2ξ*) is high enough to produce bone changes, but low enough to avoid damage, whose implications are out of the scope of the present work. On the other hand, disuse leads to drastic reductions of both *ρ*_*mat*_ and *ρ*_*app*_, completely eliminating the cortical zone. Osteoclasts remove the surplus of tissue leading to lighter but fragile bones.

In summary, the present study presents a paradigm shift offering new ways forward in the design of strategies of bone remodelling models to obtain more accurate bone structure predictions. The principal limitation of the present model is that it is analytical and so, the numerical results correspond to a macroscopic continuum model of bone that considers the distribution of the tissue in time but is averaged in space. Although we manage to capture some indirect geometrical effects through the resorption window strategy, a model of bone remodelling in 3-D, one that is capable of reproducing the specific geometry of bone trabeculae (e.g. a finite element approach) could refine these results by truly illustrating them in 3D space, thus becoming an important step forward to a better understanding of remodelling bone dynamics.

## Ethics

The density data on which this model is based was produced in previous papers in the Biomechanics laboratories of the Cranfield Forensic Institute, from specimens from an Asian Elephant which died from natural causes and provided by Whipsnade Zoo through Prof. J Hutchinson of the Royal Veterinary College of Univ. London. There was no ethical issues because the animal involved died for reasons entirely unrelated to the study and was donated without restrictions on its research usage to John Hutchinson at the RVC. As the animal remained within the UK/EU there were no IUCN Endangered species status restrictions either on transport of its tissues for such research, and of course, there were no welfare/suffering issues as the animal did not become part of the research project until well after its untimely demise.

## Supporting information

S1 Fig Apparent bone density vs ash density. **Comparison of the model output to recent data in the literature.** Measurements extracted from Schileo et al. [38] superimposed to numerical results derived from the present model for (*κ*_*sr*_ = 150). Note that the ash density is defined as the ratio of mineral mass to the reference bone volume (*ρ*_*m*_*V*_*m*_/*V*_*t*_).
(TIF)

S2 FigResults of the model by using the Specific Surface from Martin [30] ($\kappa_{sr}^{M}$) and from Adams et al.(2014) measurements ($\kappa_{sr}^{AZ}$), compared with other data from Zioupos et al.[13].**Derivation of different model outputs depending on different inputs for the bone surface density function.** Note that the smaller specific surface from Adams et al.[28] implies narrower temporal windows of resorption and a lower osteoclasts activity, translating all points to the higher material density zone. However, the boomerang-like pattern still emerges in all these cases simply shifting it position to higher or lower density values. In order to match precisely the data with specific surface values from Adams et al.[28], a wider temporal window should be implemented, meaning that with a smaller specific surface, osteoclasts may reach the inner and more mineralized bone to achieve the agreement between measurements and model results.(TIF)

S3 Fig Histomorphometry of human elephant and human femoral cancellous samples in comparison. Plots of Tb.Th, Tb.N, Tb.Sp and BS/TV versus BV/TV. The histomorphometry of these two species is very similar and thus the full ranges of samples used here allowed us to build a more generic model. This model has been validated for the present elephant samples but can be finely tuned for human or other mammalian tissues by suitable adjustment of the various parameters.
(TIF)

S1 Text Appendix to the main text describing the early evolution of the model. (DOCX)

S1 Table Values of the parameter of the model presented in supporting information. (DOCX)
