# Supplementary figures and images for: Localized tissue mineralization regulated by bone remodelling: A computational approach
Source: PLoS One. 2017 Mar 17;12(3):e0173228. doi: 10.1371/journal.pone.0173228 (PMC5357005; doi:10.1371/journal.pone.0173228)

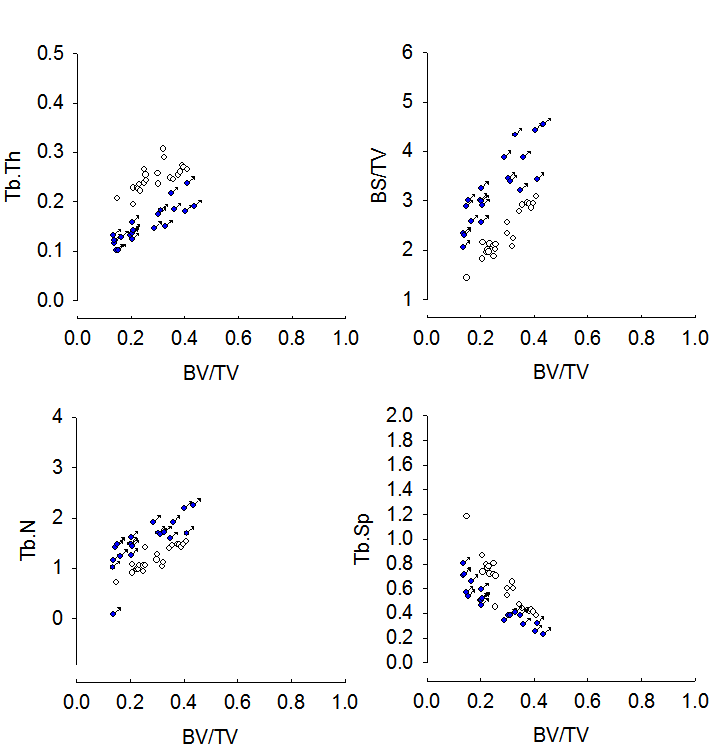

Supplement: S3 Fig — Plots of Tb.Th, Tb.N, Tb.Sp and BS/TV versus BV/TV. The histomorphometry of these two species is very similar and thus the full ranges of samples used here allowed us to build a more generic model. This model has been validated for the present elephant samples but can be finely tuned for human or other mammalian tissues by suitable adjustment of the various parameters. (TIF) [file pone.0173228.s003.tif]
