# Supplementary material for: Localized tissue mineralization regulated by bone remodelling: A computational approach
Source: PLoS One. 2017 Mar 17;12(3):e0173228. doi: 10.1371/journal.pone.0173228 (PMC5357005; doi:10.1371/journal.pone.0173228)
Supplement: S1 Text — (DOCX) [file pone.0173228.s004.docx]

**S1 Text. Appendix to the main text describing the early evolution of the model**

The evolution of bone volumes in time is governed by the Basic Multicellular Unit (BMU) activity, by resorbing bone by osteoclasts and depositing it by osteoblasts. Under normal conditions and health, bone resorption by osteoclasts is followed by osteoblastic bone formation, so that resorbed lacunae are filled to the original level by osteoblasts [43] and the bone remains with almost the same mass and structure [44]. If no regulation is exerted by osteocytes from bone matrix (inhibitory signal, s), it is believed that bone lining cells are inclined to activate BMU [45], depending on metabolic and biological factors ($f_{bio}$) and, of course, on the available free surface on which BMU may act ($S_{v}$). Thus, BMU activity is measured by its activation frequency, expressed as [20,21]:

eq S.1

The inhibitory signal $s$ from the osteocytic network follows here the theory proposed by Martin [45] and is defined as:

$s=\frac{\xi}{\xi+c}\left(1-d\right)^{a}$ eq S.2

where *a* and *c* are constants extracted from Martinez-Reina et al [21] (see S1 Table).

Osteocytes sense not only mechanical stimulus ($\xi$), but also the appearance of micro cracks due to tissue damage ($d$). For the former, the stimulus is expressed as the daily strain history based on the strain level and the number of cycles, $n_{i}$, for each load case [46]:

$\xi=\left(\sum_{i} n_{i}\bar{\varepsilon}_{i}^{m}\right)^{1/m}$ eq S.3

where the parameter $m$is taken to be 4 [46] and the effective strain, $\bar{\varepsilon}$ is defined as a function of the strain energy density ($U$), and the actual elastic modulus, $E$:

$\bar{\varepsilon}=\sqrt{\frac{2U}{E}}$ eq S.4

Damage ($d$), the other variable sensed by osteocytes, is related to density microcracks and can be measured macroscopically. It is used in the isotropic theory of continuum damage mechanics [21]. Its values are restricted to the interval [0,1], with $d=0$corresponding to an undamaged state and $d=1$to local rupture situation. Following this theory, damage may also be correlated with the mechanical degradation or loss of stiffness, as:

$d=1-\frac{E}{E_{0}}$ eq S.5

where $E_{0}$ is the elastic modulus of the bone in an ideal situation [21]. Development of the theory and detailed equations for damage and its evolution under compression or tension can be consulted in Martinez-Reina et al. [21].

The elastic modulus of the tissue evolves with the bone volume $v_{b}$ and the ash $\alpha$ fractions, and is estimated by:

$$E\left(MPa\right)=84370 v_{b}^{2.58} \alpha^{2.74} \left(1-d\right)$$

Finally the Poisson ratio is set to a constant value of $\nu=0.3$ [20]
