# Supplementary material for: Localized tissue mineralization regulated by bone remodelling: A computational approach
Source: PLoS One. 2017 Mar 17;12(3):e0173228. doi: 10.1371/journal.pone.0173228 (PMC5357005; doi:10.1371/journal.pone.0173228)
Supplement: S1 Table — (DOCX) [file pone.0173228.s005.docx]

**S1 Table. Values of the parameter of the model presented in supporting information**

| Parameter |  | Value |
| --- | --- | --- |
| *General parameters* | |  |
| *m* | Weighting exponent | 4 |
| *a* | Damage activation exponent | 40 |
| $c$ | Reference equilibrium stimulus | 0.025 |
| *f_bio_* | Biological frequency factor | 0.005 |
| $E_{0}$ | Elastic modulus of undamaged bone | 84370 MPa |
| $\nu$ | Poisson ratio | 0.3 |
